# Association Between the Severity of Depressive Symptoms and Human-Smartphone Interactions: Longitudinal Study

**DOI:** 10.2196/42935

**Published:** 2023-02-22

**Authors:** Xiao Yang, Jonathan Knights, Victoria Bangieva, Vinayak Kambhampati

**Affiliations:** 1 Mindstrong Health Menlo Park, CA United States

**Keywords:** depression, human-smartphone interaction, longitudinal data analysis, within-person effect, between-person effect, nonergodicity

## Abstract

**Background:**

Various behavioral sensing research studies have found that depressive symptoms are associated with human-smartphone interaction behaviors, including lack of diversity in unique physical locations, entropy of time spent in each location, sleep disruption, session duration, and typing speed. These behavioral measures are often tested against the total score of depressive symptoms, and the recommended practice to disaggregate within- and between-person effects in longitudinal data is often neglected.

**Objective:**

We aimed to understand depression as a multidimensional process and explore the association between specific dimensions and behavioral measures computed from passively sensed human-smartphone interactions. We also aimed to highlight the nonergodicity in psychological processes and the importance of disaggregating within- and between-person effects in the analysis.

**Methods:**

Data used in this study were collected by Mindstrong Health, a telehealth provider that focuses on individuals with serious mental illness. Depressive symptoms were measured by the Diagnostic and Statistical Manual of Mental Disorders Fifth Edition (DSM-5) Self-Rated Level 1 Cross-Cutting Symptom Measure-Adult Survey every 60 days for a year. Participants’ interactions with their smartphones were passively recorded, and 5 behavioral measures were developed and were expected to be associated with depressive symptoms according to either theoretical proposition or previous empirical evidence. Multilevel modeling was used to explore the longitudinal relations between the severity of depressive symptoms and these behavioral measures. Furthermore, within- and between-person effects were disaggregated to accommodate the nonergodicity commonly found in psychological processes.

**Results:**

This study included 982 records of DSM Level 1 depressive symptom measurements and corresponding human-smartphone interaction data from 142 participants (age range 29-77 years; mean age 55.1 years, SD 10.8 years; 96 female participants). Loss of interest in pleasurable activities was associated with app count (*γ*_10_=−0.14; *P*=.01; within-person effect). Depressed mood was associated with typing time interval (*γ*_05_=0.88; *P*=.047; within-person effect) and session duration (*γ*_05_=−0.37; *P*=.03; between-person effect).

**Conclusions:**

This study contributes new evidence for associations between human-smartphone interaction behaviors and the severity of depressive symptoms from a dimensional perspective, and it highlights the importance of considering the nonergodicity of psychological processes and analyzing the within- and between-person effects separately.

## Introduction

Depression is a multidimensional affective disorder, which often presents as an inability to experience pleasure in some or all activities, depressed mood, disturbed sleep, and fatigue or loss of energy [1]. The assessment of depression symptom severity is traditionally performed via clinical interviews or self-report questionnaires. Additionally, a new body of empirical evidence supports using passive sensing signals to explain or predict the severity of depressive symptoms [2]. We refer to these studies as “behavioral sensing” studies based on recommendations made previously [3]. The underlying premise is that passive sensing signals from low-level sensors may be indicative of high-level behavioral markers, and these high-level behavioral markers are believed to be related to different dimensions of depression (for a review of behavioral sensing, see a previous article [4]). We will briefly revisit empirical studies that examined the associations between depressive symptoms and the behavioral measures computed from passive sensing signals.

First, the inability to experience pleasure (or loss of interest) is one of the main diagnostic criteria of major depressive disorder [5]. The scale developed to measure the inability to experience pleasure assesses the extent of enjoyable experiences through a list of pleasurable activities, including domains of food/drink, pastime/interest, social interactions, and pleasurable sensory experiences [6-8]. Based on this theoretical view and measurement scale, individuals who experience a loss of interest in pleasurable activities may be less likely to leave the house, visit public places, such as restaurants and theaters, or visit friends and family. This kind of behavior has been reported in empirical studies that measured depressive symptoms using passively sensed GPS data. Nickels et al [9] reported a negative correlation between depressive symptoms and the number of unique location clusters. Meyerhoff et al [10] reported a negative correlation between depressive symptoms and the number of unique GPS locations, and a negative correlation between depressive symptoms and the entropy of time spent in each location. Entropy typically quantifies the extent to which energy is dispersed throughout a system. Zhang et al [11] reported a negative correlation between depressive symptoms and the entropy of time spent in each location. Some other studies support the importance of unique physical locations through machine learning methods. For example, Opoku Asare et al [12] reported that internet regularity, which indicates the routineness of visiting different locations, is the most important predictor of depressive symptom.

Based on the previously reported association between depressive symptoms and a lack of diversity in physical locations, we propose the possibility that loss of interest in pleasurable activities can also manifest when people interact with the digital world on their smartphones. Smartphone apps facilitate engagement in pleasurable activities (eg, engaging in social interactions, listening to music, shopping), and a lack of diversity in smartphone app usage may be used to gauge a loss of interest in pleasurable activities as a symptom of depression. Smartphone app usage as an indicator of depression symptomatology is also informed by the Use and Gratification Theory from media psychology, which suggests that an individual’s media behavior is often considered to be the product of active intentional choices to fulfill certain needs, including information seeking, relaxation, social interaction, diversion, or escape [13]. In this analogy, smartphone apps are media that allow people to interact with their environment to fulfill such needs, and this is analogous to the physical locations that allow people to fulfill specific needs (eg, social interaction, shopping, and entertainment). Therefore, we hypothesized that behavioral measures that describe app usage, such as the number of unique apps (or app count) and entropy of time spent in each app (or app entropy), will be related with depressive symptom measures such as a lack of interest in pleasurable activities.

Second, depressed mood, which refers to feeling sad, helpless, and hopeless, is another important diagnostic criterion for depression. It has been suggested that it is challenging for passive sensing signals to assess mood consistently and accurately since mood is more distal from the sensors and features normally used in behavioral sensing (with the exception of recording of speech; for a review, see a previous article [4]). If we zoom out to depressive symptoms, instead of focusing on depressed mood specifically, there is some empirical evidence to suggest that depressive symptoms may be associated with sleep duration, physical activity, heart rate, and social interaction [14-17]. A research study reported that longer sleep durations computed from wearable devices, in conjunction with ecological momentary assessments of emotions (arousal and valence), as predictors were associated with higher depressive symptoms as the outcome [14]. Narziev et al [15] reported that physical activity and heart rate measured via passive sensing can predict depressive symptoms. Bai et al [16] reported that call logs (social interaction), step count (physical activity), heart rate, and sleep measured via passive sensing can predict the stability of mood. Jacobson et al [17] reported actigraphy (physical movement) to be associated with depressive symptoms. In some of these studies, a machine learning approach was used and the directionality between depressive symptoms and passive sensing signals was unspecified [14,16,17]; thus, it is challenging to know the specific associations between depressed mood and measures computed from passive sensing.

Lastly, sleep disruption and psychomotor functioning that have been computed from passive sensing measurements have been suggested to be associated with depressive symptoms. For example, insomnia is a common symptom of depression (hypersomnia, or excessive sleep, can also be a symptom of depression), and phone use (both count and duration) during sleep windows has been reported to be related to poor subjective sleep quality [18]. Further, Giancardo et al [19] suggested that it is feasible to detect psychomotor impairment due to sleep inertia via finger interactions with a computer keyboard during natural typing. Vesel et al [20] reported that keystroke dynamics, including slower typing speed, higher typing speed variability, shorter session duration, and lower accuracy, were associated with more severe depression. Zulueta et al [21] reported that higher average interkey delay (slower typing speed) was associated with more severe depressive symptoms. Based on these findings, we hypothesize that behavioral measures that are related to sleep disruption and psychomotor functioning will be related with depressive symptom measures.

In summary, in this work, we explore the association between self-reported depressive symptoms and human-smartphone interaction behaviors based on theoretical propositions and previous empirical evidence introduced. Specifically, we build behavioral measures that we believe are indicative of a loss of interest in pleasurable activities (eg, lower app count and lower app entropy), sleep disruption (eg, higher phone usage during nighttime), and psychomotor functioning (eg, slower typing speed and shorter session duration) from passively sensed human-smartphone interaction data, and we hypothesize that these measures will be associated with a loss of interest in pleasurable activities and depressed mood. In order to accommodate the nested nature of repeated longitudinal data (self-report and behavioral measurements nested within persons), hypotheses were examined within a multilevel modeling framework [22,23]. Following the recommended practice [24], the predictor variables were split into within-person changes (time-varying component) and between-person differences (time-invarying component), and entered into the multilevel model as separate predictors. The between- and within-person associations between behavioral measures and depressive symptom measures will be examined and used to identify behavioral characteristics that are associated with higher levels of depressive symptoms.

## Methods

### Participants

In this retrospective observational study, 142 patients who received virtual mental health care at Mindstrong Health (Mindstrong) were included. Mindstrong is a telehealth provider that specialized in serious mental illness and uses passive sensing technology through a mobile app to inform treatment. The age of patients in this sample ranged from 29 to 77 years, with a mean of 55.1 (SD 10.8) years. Of the 142 patients, 96 (67.6%) were female, 43 (30.3%) were male, and 3 (2.1%) were unidentified. Moreover, 87 patients (61.3%) were White, 11 (7.8%) were African American, 6 (4.2%) were Hispanic/Latino, and 38 (26.8%) were unidentified. These patients received treatment at Mindstrong Health between October 2020 and September 2021. The primary diagnosis was determined by licensed mental health providers at Mindstrong at the start of treatment. In this sample, there were 83 patients (58.5%) with a primary diagnosis of major depressive disorder, 45 (31.7%) with a primary diagnosis of bipolar disorder, and 14 (9.9%) with a primary diagnosis of schizophrenia. These patients were from 11 different states in the United States.

### Procedure

All patients installed the Mindstrong Health app and provided informed consent for the use of data in research and product development before enrolling for services. This analysis was conducted under a secondary data analysis protocol to identify clinically relevant associations in active and passive data collection at Mindstrong.

**Figure 1:**
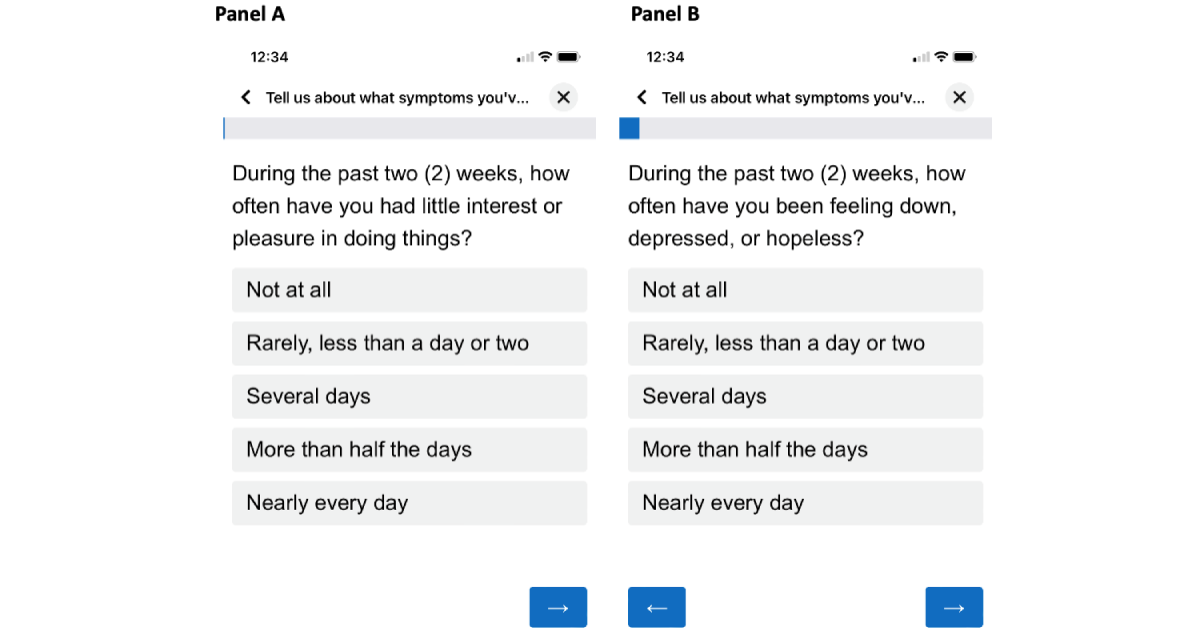
Screenshots of the format of the Diagnostic and Statistical Manual of Mental Disorders Fifth Edition (DSM-5) Self-Rated Level 1 Cross-Cutting Symptom Measure-Adult Survey (DSM L1) questionnaire that was delivered to patients via the Mindstrong app. Panels A and B are the 2 items in the DSM L1 that represent the domain of depression. Panel A shows item 1 in the DSM L1 for measuring loss of interest in pleasurable activities, and Panel B shows item 2 in the DSM L1 for measuring depressed mood.

As part of routine clinical care, patients were asked to report their mental illness symptoms every 60 days through the mobile app via the Diagnostic and Statistical Manual of Mental Disorders Fifth Edition (DSM-5) Self-Rated Level 1 Cross-Cutting Symptom Measure-Adult Survey (DSM L1). Due to possible patient burden given the serious mental illness population, the DSM L1 was chosen as a routine clinical screener to comprehensively assess a wide range of clinical domains while ensuring assessment brevity. Specifically, the DSM L1 consists of 23 questions that assess 13 clinical domains, including depression, anger, mania, anxiety, somatic symptoms, suicidal ideation, psychosis, sleep problems, memory, repetitive thoughts and behaviors, dissociation, personality functioning, and substance use (on average, 1-3 items are present per clinical domain) [25]. The depression domain in the DSM L1 has 2 items, which assess loss of interest in pleasurable activities and depressed mood (feeling sad). These 2 items are comparable to the short-form Patient Health Questionnaire (PHQ2) [26]. The DSM L1 questionnaire was completed by patients every 60 days via the Mindstrong app. Screenshots of the DSM L1 survey in the Mindstrong app are shown in Figure 1. A total of 984 assessments of depressive symptoms over a 1-year period were included in this analysis.

In this retrospective observational study, patients were selected based on high compliance with the DSM L1. We intentionally selected individuals with higher compliance because higher compliance (or more repeated measures within the same person) allows examination of the within-person association between self-reports of depressive symptoms and the behavioral measures computed from human-smartphone interactions.

**Figure 2:**
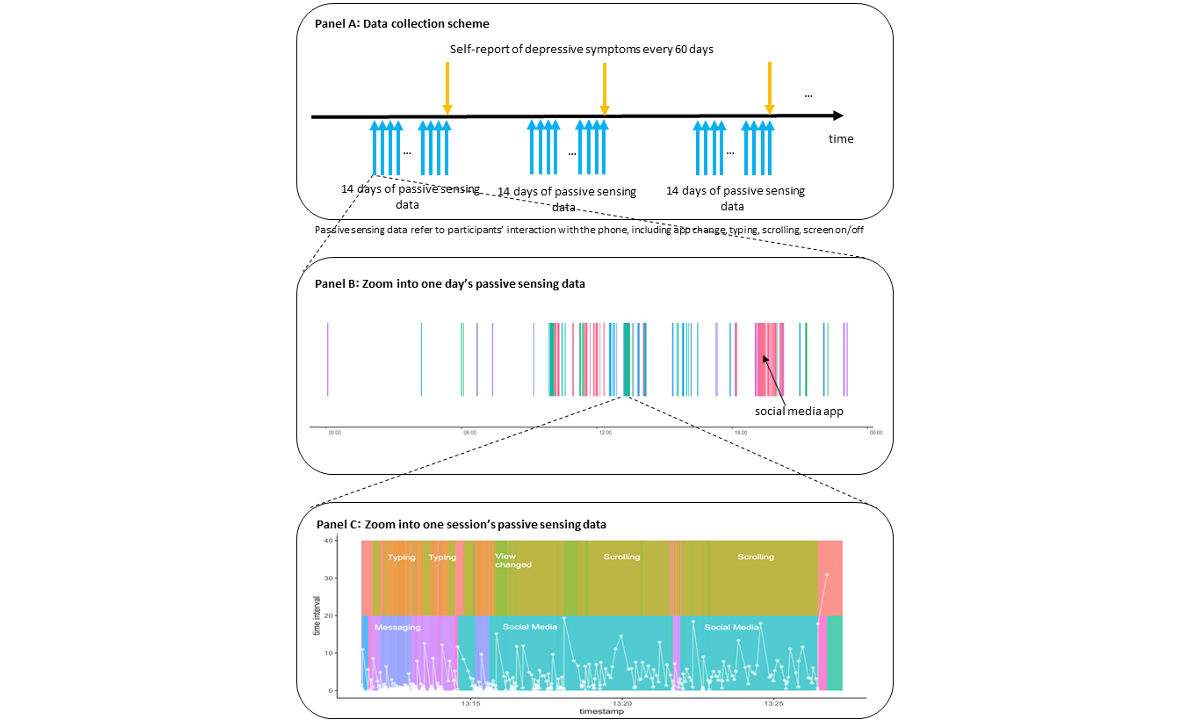
Illustration of the procedure and measurements. Panel A shows the data collection scheme described in the procedure, with alignment of the 14 days of passive sensing data prior to each depressive symptom self-report. Panel B shows an example of a day’s passive sensing data, where each color represents a unique app and its area represents the time spent in that app (we marked the color of the social media app where this person spent more time than other apps). Panel C shows an example of a session’s passive sensing data (session is defined as a sequence starting from screen on and ending at the next screen off), where the top row shows events of typing, clicking, scrolling, and view change marked by different colors, and the bottom shows apps by different colors (to preserve the privacy of this participant, we only labeled messaging and social media to demonstrate the general categories of the apps). The white lines show time elapsed for a specific interaction event.

Because the DSM L1 prompted participants to report their depressive symptoms that occurred during the past 2 weeks (or 14 days), we included 14 days of smartphone interaction data prior to each depressive symptom measurement date (symptom-date) in the analysis. The temporal alignment between the DSM L1 survey and smartphone metadata is shown in Figure 2A.

The app collects smartphone metadata and contains information about interactions with smartphones in an unobtrusive manner. These metadata of device usage and its touchscreen are collected unobtrusively by proprietary software on the Android operating system. The metadata include various touchscreen behaviors (eg, clicking and scrolling), device-level behaviors (eg, turning the smartphone screen on and turning the smartphone screen off), masked keyboard behaviors (eg, typing characters from the left or right side of the smartphone’s keyboard and not the exact character), and change of foreground apps (eg, text messaging apps and entertainment apps). The starting time of each instance of usage of the smartphone device and its touchscreen was recorded with a timestamp at the millisecond level. These metadata are collected locally on the patient’s smartphone and are then transmitted with encryption to a Health Insurance Portability and Accountability Act (HIPAA)– and ISO 27001–compliant cloud storage service (Amazon Web Services). All personnel who can access patients’ metadata and assessment data (including diagnosis, demographics, and self-reported survey data) complete annual HIPAA training. Data were deidentified prior to analysis.

### Ethics Approval

The analysis has been reviewed and approved as exempt by the WCG Institutional Review Board (Puyallup, WA).

### Measurements

#### Depressive Symptoms

Depressive symptoms were assessed by 2 items in the DSM L1 self-report questionnaire [25], which are comparable to the PHQ2 [26]. The DSM L1 is a 23-item questionnaire that assesses how much or how often the responder is bothered by symptoms across 13 clinical domains. For this analysis, we only used responses to the depression domain, which has 2 questions (Figure 1). Both questions contain the prompt “during the past 2 weeks.” Item 1 measures loss of interest, which prompts “how often have you had little interest or pleasure in doing things?” Item 2 measures depressed mood, which prompts “how often have you been feeling down, depressed, or hopeless?” Participants select their responses in the app using a 5-point Likert scale (0=not at all/none; 1=rare, less than a day or two/slight; 2=several days/mild; 3=more than half the days/moderate; 4=nearly every day/severe).

#### App Count

App count was defined as the number of unique foreground apps present during an hour. This measure is intended to capture the variety of a person’s activity on their smartphone, such as information seeking, entertainment, and social interaction [12]. An example of a day’s app usage is illustrated in Figure 2B, where each color represents a unique app.

#### App Entropy (Normalized)

App entropy captures the variability and complexity of app use during a given hour [12]. Higher app entropy is indicative of more evenly distributed allocation of time in each app, whereas lower app entropy indicates more unequally distributed and potentially concentrated use of apps. App entropy was defined as a normalized version of Shannon entropy of foreground app behavior as follows:



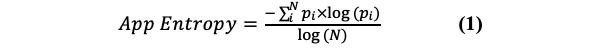



where is the proportion of time spent on a specific app out of the total time of using all the apps and *N* in the given hour. To avoid bias in the entropy measure from the number of unique apps (ie, higher number of apps will have higher app entropy), we normalized the app entropy by dividing the logarithm of the total number of unique apps. An example of a day’s worth of data is illustrated in Figure 2B, where each color represents a different app (eg, the social media app is marked by magenta). Spending the most time in 1 app within a given hour produces a lower app entropy compared to a more evenly distributed app usage.

#### Nighttime Smartphone Use

To measure phone-related sleep disruption during periods of presumed sleep, we used human-smartphone interactions from midnight (12 AM) to 6 AM to approximate the sleep window and summed up the active screen time over these 6 hours each day. In the example of a day’s worth of app usage illustrated in Figure 2B, we can observe that there is considerable white space, indicating no use of the smartphone device. On the other hand, there was sporadic nighttime phone use (marked by purple and green bars in Figure 2B) between midnight and 6 AM.

#### Session Duration

A smartphone session measures the length of a sequence of smartphone activity. This measure was defined as the time from when the smartphone screen is turned on to the time when the smartphone screen is turned off [20]. Because time-based variables are often exponentially distributed and the median is a more accurate summary statistic than the mean, we chose the median to represent the session duration within an hour. An example session is illustrated in Figure 2C, where a sequence of interaction events occurred, including typing, view change, scrolling, and app change from messaging to social media.

#### Typing Speed

The time interval of typing behavior has been suggested to measure processing speed as an indicator of psychomotor functioning [19-21]. Typing behavior on smartphones includes events when individuals use their keyboard to type characters, numbers, or other keys (eg, delete and backspace). The time interval of typing behavior was computed as the time difference from the starting time of a typing event to the starting time of the next event. This typing behavior often occurs on the millisecond timescale. To avoid abnormally large values, which could be due to being distracted from typing, we capped the time interval of typing behavior to 5 seconds (5000 milliseconds). In the example session illustrated in Figure 2C, the typing time interval is illustrated by the white line when typing events occurred.

### Data Preprocessing

#### Selection Criteria

Previously published literature for passively sensed behavioral measures [9] has suggested that the passive sensing data within a particular time frame need to be present in sufficient quantities to rule out cases where sensors were not collecting data in a continuous fashion for some reason (eg, criteria of 3 days out of a week and 18 unique hours per day [9]). Because human-smartphone interaction sensing will only produce data when participants are actively interacting with the phone, obtaining active data for 24 hours is unlikely; therefore, we relaxed the selection criteria for passive sensing data to at least 3 unique hours per day and at least 3 unique days within the 14-day window per DSM L1 measurement.

As a result, the sample that was entered into the multilevel modeling analysis had 142 participants and 984 measurements of depressive symptoms, where each assessment of depressive symptoms had at least 3 different days and 3 different hours of phone interaction data per day prior to the DSM L1 measurement (totaling 12,098 person-days and 196,673 person-hours).

#### Skewness and Outliers

Owing to the skewness in the distribution of time-related measures (eg, nighttime phone use, session duration, and typing time interval), logarithm transformation was applied to the data as a preprocessing approach. Since typing often occurs on the millisecond timescale and there are outliers when the typing time interval is extreme, potentially due to malfunction of the phone or distraction when typing, we capped the typing time interval at 5 seconds [20].

#### Aggregation of Behavioral Measures to Align With Depressive Symptoms

We computed the mean of each behavioral measure described in the Measurements section for the 14 days preceding each DSM L1 measurement, so that we could align the behavioral measures as predictors and depressive symptoms as outcomes in the multilevel model.

### Analysis Plan

In order to accommodate the nested nature of repeated longitudinal data (984 self-report and behavioral measurements nested within 142 persons), hypotheses were examined within a multilevel modeling framework [22,23]. In longitudinal data analysis, it is highly recommended to disaggregate 2 types of contributing factors, namely the within- and between-person effects [24]. *Within-person effects* refer to the effects of the time-varying portion of a predictor on the outcome, which means the association between the outcome and the changes when comparing a person with themselves across time points. *Between-person effects* refer to the effects of the time-invarying portion of a predictor (eg, trait-like stable characteristic) on the outcome, which means the association between the outcome and the differences when comparing one person with another person [24]. The reason to disaggregate the within- and between-person effects is that psychological processes are often nonergodic, which means the between-person effects are rarely the same as the within-person effects [27]. These 2 types of effects explain different mechanisms, for example, people *who* have a consistent trait-like low app count might have personal phone use habits that have very little to do with depression, but *when* people suddenly use much fewer apps, they might have a loss of interest in pleasurable activities, and this can indicate a risk of elevated depressive symptoms.

We tested the associations between human-smartphone interaction behaviors and depressive symptoms with respect to loss of interest in pleasurable activities and depressed mood separately. Thus, 2 multilevel models were tested, where the first model used loss of interest in pleasurable activities as the dependent variable, and the second model used depressed mood as the dependent variable, while the predictors remained the same. Relations among the extended set of variables were then examined using 2-level models of the following form:

{*Loss_of_Interest_it_*, *Depressed_Mood_it_*} = *β*_0_*_i_* + *β*_1_*_i_* × *wp.AppCount_it_* + *β*_2_*_i_* × *wp.AppEntropy_it_* + *β*_3_*_i_* × *wp.NighttimePhoneUse_it_* + *β*_4_*_i_* × *wp.SessionDuration_it_* + *β*_5_*_i_* × *wp.TypingInterval_it_* + *e_it_*
**(2)**

*β*_0_*_i_* = *γ*_00_ + *γ*_01_ × *bp.AppCount_i_* + *γ*_02_ × *bp.AppEntropy_i_* + *γ*_03_ × *bp.NighttimePhoneUse_i_* + *γ*_04_ × *bp.SessionDuration_i_* + *γ*_05_ × *bp.TypingInterval_i_* + *u*_0_*_i_*
**(3)**

*β*_1_*_i_* = *γ*_10_ + *u*_1_*_i_*
**(4)**

*β*_2_*_i_* = *γ*_20_ + *u*_2_*_i_*
**(5)**

*β*_3_*_i_* = *γ*_30_ + *u*_3_*_i_*
**(6)**

*β*_4_*_i_* = *γ*_40_ + *u*_4_*_i_*
**(7)**

*β*_5_*_i_* = *γ*_50_ + *u*_5_*_i_*
**(8)**

where the repeated measures of loss of interest in pleasurable activities (shortened as loss of interest in the equation for brevity) or depressed mood for individual *i* at time *t*, *Loss_of_Interest_it_* or *Depressed_Mood_it_*, were modeled as a function of person-specific intercepts, *β*_0_*_i_*, that indicate the baseline level of depressive symptoms, and person-specific coefficients, *β*_1_*_i_* to *β*_5_*_i_*, that indicate the extent of within-person associations between human-smartphone interaction behaviors and depressive symptoms. *γ_00_* to *γ_50_* are sample-level parameters. *u*_0_*_i_* to *u*_5_*_i_* are the residual unexplained between-person differences and are assumed to be multivariate normal with mean equal to zero and variances 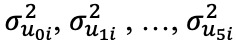 .

The model was fit to the data using the *lme4* package in R (version 1.1-29; R Project for Statistical Computing) [28], with incomplete data (0.2%) treated as missing at random. Person-specific intercepts and coefficients were simultaneously modeled as functions of the between-person and within-person portions of the predictors, respectively.

There is a tradeoff between the parsimoniousness of the model, especially the covariance structure of the random effects and the goodness of fit to the data. For our purpose to test the association between human-smartphone interaction behaviors and depressive symptoms, it was important to include a random slope of within-person predictors to allow for the heterogeneous person-specific association between human-smartphone interaction behaviors and depressive symptoms. However, this study did not focus on the covariance of random slopes. Therefore, we chose an independent structure of the random effects to have a parsimonious model and have the capacity to model the random slopes.

## Results

### Summary Statistics of the Measurements

Summary statistics of the behavioral measurements of human-smartphone interactions, including mean, SD, minimum, maximum, and skewness, and the correlations with loss of interest in pleasurable activities or depressed mood are shown in Table 1. After log transformation, the skewness of the behavioral measures (nighttime phone use, session duration, and typing time interval) reduced to less than 1. The severity of loss of interest in pleasurable activities was at a mean of 2.24 (SD 1.21), and that of depressed mood was at a mean of 2.08 (SD 1.24).

The correlations between behavioral measures and loss of interest in pleasurable activities or depressed mood are also shown in Table 1. To demonstrate the difference in disaggregating within- and between-person portions of the predictors, we included all 3 types of correlations, namely the correlation between loss of interest in pleasurable activities (or depressed mood) and the raw predictor (without disaggregating the within- and between-person portions), the correlation between loss of interest in pleasurable activities (or depressed mood) and the within-person portion of the predictor, and the correlation between loss of interest in pleasurable activities (or depressed mood) and the between-person portion of the predictor. After disaggregating the within- and between-person portions, the correlation between the outcome (loss of interest in pleasurable activities or depressed mood) and the predictors (human-smartphone interaction behaviors) was different from the correlation between the outcome and either the within-person portion or the between-person portion (Table 1). For example, loss of interest in pleasurable activities had a correlation of −0.01 with raw app count, but it had a correlation of −0.07 with the within-person portion of app count and a correlation of 0.02 with the between-person portion of app count.

**Table 1:** Data of human-smartphone interaction behaviors and their correlations with loss of interest in pleasurable activities and depressed mood.

Measure	Value			Skewness		Correlation with loss of interest in pleasurable activities				Correlation with depressed mood			
	Mean (SD)	Range (minimum-maximum)			Raw		Within	Between	Raw		Within	Between	
App count	7.53 (2.06)	2.20-15.55	0.76		−0.01		−0.07	0.02	−0.02		−0.04	−0.005	
App entropy normalized	0.48 (0.09)	0.20-0.70	−0.34		−0.01		−0.0009	−0.007	−0.02		−0.009	−0.02	
Nighttime phone use^a,b^	1.70 (1.36)	0.00-5.27	0.57/2.31^c^		0.03		0.02	0.02	−0.02		−0.02	−0.02	
Session duration^a,b^	1.35 (0.67)	0.19-4.11	0.63/2.20^c^		−0.09		−0.02	−0.09	−0.12		−0.02	−0.12	
Typing time interval^a,d^	0.43 (0.15)	0.00-1.03	0.64/4.76^c^		−0.02		0.06	−0.04	−0.03		0.05	−0.05	

^a^Log transformed.

^b^The unit is minutes.

^c^Skewness before log transformation.

^d^The unit is seconds.

### Associations Between Loss of Interest in Pleasurable Activities and Human-Smartphone Interaction Behaviors

**Figure 3:**
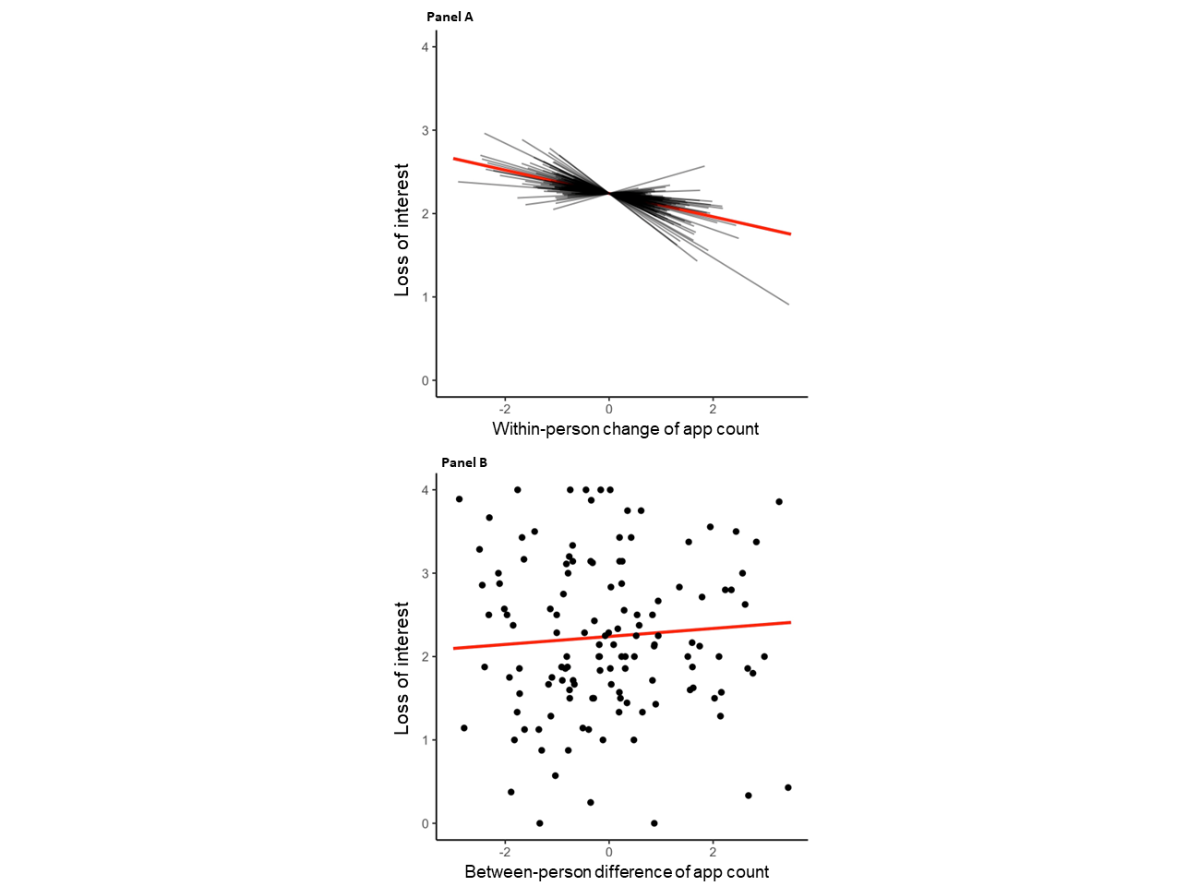
The within- and between-person associations between app count and loss of interest in pleasurable activities. Panel A shows the within-person associations, with the sample-level estimated slope of within-person change of app count in red and the person-level estimated slope of within-person change of app count in black. Panel B shows the between-person associations, with the sample-level estimated slope of between-person difference of app count in red and a scatter plot of the person-mean of app count and person-mean of loss of interest in pleasurable activities.

In the results of model 1 (details shown in Tables 2 and 3), the level of loss of interest for a prototypical individual was *γ*_00_=2.24 (*P*<.001) on a scale of 0 to 4. The within-person association between loss of interest in pleasurable activities and app count was *γ*_10_=−0.14 (*P*=.01). This indicates that when comparing a participant’s app count with their own average app count, if there was a decrease in app use, this person had a higher level of loss of interest in pleasurable activities. The random effect of this association indicates the variance of this association across participants (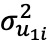
=0.06). This variance is visualized in Figure 3A as heterogeneous associations between the within-person change in app count and loss of interest in pleasurable activities. On the other hand, the between-person association between loss of interest in pleasurable activities and app count was *γ*_01_=0.05 (*P*=.51), and this indicates that participants who had a lower app count did not have a higher level of loss of interest in pleasurable activities. The between-person association is visualized in Figure 3B.

The within-person association between normalized app entropy and loss of interest in pleasurable activities was *γ*_20_=1.35 (*P=*.18), and the between-person association was *γ*_02_=−1.64 (*P*=.35). This indicates that changes in normalized app entropy were not associated with different levels of loss of interest in pleasurable activities, either when comparing a person with themselves or comparing a person with others.

The within-person association between nighttime phone use and loss of interest in pleasurable activities was *γ*_30_=0.07 (*P*=.10), and the between-person association was *γ*_03_=0.06 (*P*=.45). This indicates that changes in nighttime phone use were not associated with different levels of loss of interest in pleasurable activities, either when comparing a person with themselves or comparing a person with others.

The within-person association between session duration and loss of interest in pleasurable activities was *γ*_40_=0.08 (*P*=.49), and the between-person association was *γ*_04_=−0.29 (*P*=.07). This indicates that changes in session duration were not associated with different levels of loss of interest in pleasurable activities, either when comparing a person with themselves or comparing a person with others.

The within-person association between typing time interval and loss of interest in pleasurable activities was *γ*_50_=0.88 (*P*=.08), and the between-person association was *γ*_05_=−0.34 (*P*=.58). This indicates that changes in typing time interval were not associated with different levels of loss of interest in pleasurable activities, either when comparing a person with themselves or comparing a person with others.

**Table 2:** Fixed effect results from the multilevel model examining the association between depressive symptoms and human-smartphone interaction behaviors.

Parameter^a^	Model 1^b^				Model 2^c^		
	Estimate	SE^d^	*P* value	Estimate		SE^d^	*P* value
Intercept, *γ*_00_	2.24	0.08	<.001	2.09		0.08	<.001
wp.AppCount, *γ*_10_	−0.14	0.05	.01	−0.03		0.05	.57
wp.AppEntropy, *γ*_20_	1.35	1.01	.18	−0.20		1.06	.85
wp.NighttimePhoneUse, *γ*_30_	0.07	0.04	.10	−0.02		0.04	.62
wp.SessionDuration, *γ*_40_	0.08	0.12	.49	−0.03		0.12	.80
wp.TypingInterval, *γ*_50_	0.88	0.47	.08	0.88		0.44	.05
bp.AppCount, *γ*_01_	0.05	0.07	.51	0.05		0.08	.50
bp.AppEntropy, *γ*_02_	−1.64	1.74	.35	−1.98		1.82	.28
bp.NighttimePhoneUse, *γ*_03_	0.06	0.07	.45	0.02		0.08	.75
bp.SessionDuration, *γ*_04_	−0.29	0.16	.07	−0.37		0.17	.03
bp.TypingInterval, *γ*_05_	−0.34	0.60	.45	−0.42		0.63	.50

^a^A total of 982 repeated measures nested within 142 persons.

^b^The dependent variable is loss of interest in pleasurable activities.

^c^The dependent variable is depressed mood.

^d^SE: standard error for fixed effects.

**Table 3:** Random effect results from the multilevel model examining the association between depressive symptoms and human-smartphone interaction behaviors.

Parameter^a^	Model 1^b^			Model 2^c^	
	Estimate	CI^d^	Estimate		CI^d^
Variance of intercept, 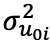	0.86	0.06-1.09	0.95		0.74-1.24
Variance of random slope wp.AppCount, 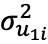	0.06	0.00-0.13	0.05		0.00-0.12
Variance of random slope wp.AppEntropy, 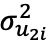	14.81	0.00-38.86	26.30		0.34-53.54
Variance of random slope wp.NighttimePhoneUse, 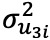	0.003	0.00-0.05	0.00		0.00-0.09
Variance of random slope wp.SessionDuration, 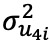	0.16	0.00-0.43	0.03		0.00-0.42
Variance of random slope wp.TypingTimeInterval, 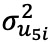	0.84	0.00-6.48	0.00		0.00-4.53
Variance of residual, 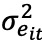	0.54	0.47-0.59	0.55		0.47-0.59

^a^A total of 982 repeated measures nested within 142 persons.

^b^The dependent variable is loss of interest in pleasurable activities.

^c^The dependent variable is depressed mood.

^d^CI: 95% CI for random effects.

### Associations Between Depressed Mood and Human-Smartphone Interaction Behaviors

In the results of model 2 (details shown in Tables 2 and 3), the level of depressed mood for a prototypical individual was *γ*_00_=2.09 (*P*<.001) on a scale of 0 to 4. The within-person association between app count and depressed mood was *γ*_10_=−0.03 (*P*=.57), and the between-person association was *γ*_01_=0.08 (*P*=.50). This indicates that changes in app count were not associated with different levels of depressed mood, either when comparing a person with themselves or comparing a person with others.

The within-person association between normalized app entropy and depressed mood was *γ*_20_=−0.20 (*P*=.85), and the between-person association was *γ*_02_=−1.98 (*P*=.28). This indicates that changes in normalized app entropy were not associated with different levels of depressed mood, either when comparing a person with themselves or comparing a person with others.

The within-person association between nighttime phone use and depressed mood was *γ*_30_=−0.02 (*P*=.62), and the between-person association was *γ*_03_=0.08 (*P*=.75). This indicates that changes in nighttime phone use were not associated with different levels of depressed mood, either when comparing a person with themselves or comparing a person with others.

The within-person association between session duration and depressed mood was *γ*_40_=−0.03 (*P*=.80), and the between-person association was *γ*_04_=−0.37 (*P*=.03). This indicates that individuals who generally had shorter sessions had a higher level of depressed mood.

The within-person association between typing time interval and depressed mood was *γ*_50_=0.88 (*P*=.047), and the between-person association was *γ*_05_=−0.42 (*P*=.50). This indicates that an increase in typing time interval (slowing down of typing speed) was associated with a higher level of depressed mood, when comparing a person with themselves. However, individuals who generally typed more slowly did not have a higher level of depressed mood.

## Discussion

### Principal Findings

In this paper, we proposed a theoretical linkage between human-smartphone interaction behaviors and depressive symptoms, suggested disaggregating the within- and between-person effects in longitudinal analysis, and analyzed the association between 142 individuals’ 984 depressive symptom measurements and their human-smartphone interaction behaviors over a 1-year period. The results demonstrated that a within-person decrease in the diversity of app use was associated with loss of interest in pleasurable activities, and a within-person decrease in typing speed (or increase in typing time interval) and a between-person difference in session duration (eg, shorter session duration) were associated with higher depressed mood.

### Understanding Depression From Smartphone Behavior

We found that loss of interest in pleasurable activities, a key diagnostic criterion for depression, was associated with within-person changes in app count, which confirmed our hypothesis that the extent of loss of interest can manifest in individuals’ use of their smartphones to fulfill various purposes and needs. Human-smartphone interaction behavior, specifically how people use apps to fulfill their needs, can serve as a behavioral measurement to measure depressive symptoms. We found that loss of interest in pleasurable activities was in the same direction but did not reach significance regarding higher nighttime phone use (*γ*_30_=0.07; *P*=.10). The direction is consistent with the findings in previous literature [18] where phone usage during sleep windows has been reported to be related to poor subjective sleep quality. Additionally, we found that loss of interest in pleasurable activities was in the same direction but did not reach significance regarding shorter smartphone session duration and longer typing time interval (slower typing speed). The direction is consistent with the findings in previous literature [20] where depressive symptoms have been reported to be associated with a shorter duration of typing sessions and slower typing speed. Lastly, in previous literature, the entropy of unique physical locations was found to be negatively associated with depressive symptoms [10,11], and this could be due to spending most of the time at home and avoiding visiting locations that used to be enjoyable. However, we did not find a significant association between the entropy of unique app use and loss of interest, which implies that more concentrated use of one or a few specific apps is not indicative of loss of interest. Future analysis could further investigate the similarities and differences in visiting physical locations versus digital applications, as it relates to their associations with loss of interest in pleasurable activities.

Depressed mood, another key diagnostic criterion for depression, was expected to be challenging to predict from human-smartphone interaction behaviors based on previous literature [4,18]. We found that a higher level of depressed mood was associated with behavioral measures that are indicative of worse psychomotor functioning, including longer typing time intervals and shorter duration of smartphone sessions. The empirical relation between mood and cognitive functioning has been reported by previous empirical studies for older adults [29]. However, it would be more direct and compelling to test the linkage between these behavioral measures and psychomotor functioning or difficulty with concentration. Since the DSM L1 used in this study did not include assessments of difficulty with concentration, it did not allow us to test this association. For studies that have such symptom measurements, for example, Patient Health Questionnaire (PHQ) [30], we recommend testing the linkage between this symptom and behavioral measures of difficulty with concentration directly.

In summary, these findings highlight the utility of passive sensing data, especially passive sensing of human-smartphone interaction, as a continuous monitoring tool for depression and potentially as a just-in-time intervention tool when elevated depressive symptoms are detected. Moreover, the findings suggest that taking a dimensional perspective of depression may inform the interpretability of behavioral measures from passive sensing and improve the precision of assessing specific depressive symptoms.

### Analyzing Depression as a Longitudinal Process

We emphasized the recommended practice of separating within- and between-person effects in behavioral longitudinal analysis due to nonergodicity in psychological processes. The analysis provided evidence that these 2 effects indeed differ with respect to depressive symptoms and provided empirical evidence that the behavioral process regarding the association between the development of depression and human-smartphone interactions can be nonergodic. With app count as an example to further elaborate on this, the within-person association between app count (number of unique apps being used) and loss of interest was significant and had a negative value, but the between-person association between the same behavioral measure and loss of interest was not significant and had a positive value. From this result, we can infer that only when people use fewer apps compared to themselves, it would indicate a risk of a higher loss of interest, but among those who use fewer apps habitually compared to others, it would not indicate a risk of a higher loss of interest. This nonergodicity was found in not only app count but also other predictors in our analysis, such as session duration (only the between-person association was significantly associated with depressed mood) and typing time interval (only the within-person association was significantly associated with depressed mood). These findings highlight the importance of disaggregating the within- and between-person effects to draw accurate statistical inferences in longitudinal studies.

There are 2 major types of risks of not following this recommended practice involving mixing within- and between-person portions as 1 predictor. First, there is a risk of identifying false-positive cases when the within- and between-person portions of the same predictor are entered into the model as a single measurement. Hypothetically, if there was a significant negative association between app count and loss of interest, without separating the between- and within-person portions, we would have misidentified people at risk for depression from those who habitually have a lower app count. Second, there is a risk that a significant predictor might be overlooked because the correlation between the outcome and the predictors can paint a murky picture before the predictors are split into between- and within-person portions. This occurs often when the within- or between-person association differs in direction, which is a specific form of nonergodicity. For example, the app count had a negative within-person association (*γ*_10_=−0.15) and a positive between-person association (*γ*_01_=0.05), but the correlation between raw app count and loss of interest was −0.01 (Table 1). Considering that the correlation between the outcome and behavioral measures is often reported in behavioral sensing studies and used as a feature selection criterion, we recommend that the correlation or other statistical associations (eg, in a multilevel model framework) be computed separately for the within- and between-person portions of the predictors in longitudinal studies and recommend the use of the respective correlation as a feature selection criterion if applicable.

In the behavioral sensing literature, the modeling approach that examines the longitudinal data of depressive symptoms and the associations with passive sensing signals often overlooks disaggregating the within- and between-person effects. It is important to point out that because of the nonergodicity in psychological processes, differentiating within- and between-person effects is a modeling choice that applies to not only statistical inference (eg, multilevel modeling) but also machine learning methods, where time-varying predictors have within- and between-person portions, and they would have distinctive associations with the outcome variable.

### Limitations

The results of this retrospective study must be interpreted with respect to some limitations. First, we built behavioral measures using passive sensing data from our proprietary software, which are expected to capture components of depressive symptoms. There are other platforms and software that aim to conduct behavioral sensing or digital phenotyping studies, such as the Beiwe system [31], LAMP [32], Effortless Assessment of Risk States system (EARS) [33], and BiAffect app [21]. It is currently a common practice in behavioral sensing studies to have measures or features created based on the researchers’ choices of software or platforms, as well as the availability and characteristics of the data. Concerns have been raised in the behavioral sensing literature regarding the generalizability of such idiosyncratic development of measurements in empirical studies [34,35]. Considering the heterogeneity that exists in data collection platforms/software, it would be challenging to ensure that the data source from every study conforms to the same data collection method or standard. Rather, it might be more realistic and reasonable to find common ground in ensuring that the behavioral measures across studies share the same conceptual meaning and are comparable. As an initial step, we aimed to enhance the replicability of this study through a few approaches, including stating explicitly the conceptual meaning of the measures, the timescale of behavioral measures (eg, hourly), and the computation method (eg, equation of app entropy), and reporting the statistics summary (eg, mean, SD, range, and skewness). Further steps of enhancing the generalizability of these measures could examine the psychometric characteristics of behavioral measures, such as reliability and validity. Additionally, it is worth noting there are other behavioral measures that have been suggested to correlate with depressive symptoms, which were not explored, including social interactions [16], physiological or movement data often collected from wearables [36], and total daily screen time [37] (we used the sum of screen time between midnight and 6 AM every day as a measure of sleep disruption).

Second, the participants in this study were older adults with serious mental illness. The characteristics of older adults’ human-smartphone interaction behaviors might not be the same as those of younger adults or adolescents. Additionally, clinical information that might have affected human-smartphone interaction (eg, dementia and vision impairment) was not collected as part of routine care at Mindstrong; thus, we cannot rule out that medical comorbidities might have been present in this sample and might have affected the behavioral metrics. Considering the history of mental illness and its influence on behavior, it is understudied whether people who have been depressed have the same behavioral characteristics as those who have not been depressed before. Further comparisons are needed to enhance the generalizability of the findings in this study, especially comparisons between older and younger adults and between a clinically depressed sample and a subacute or nonclinically depressed sample.

Third, the appropriate timescale of behavioral measures has been rarely discussed in behavioral sensing literature, and it is unclear how shorter to longer timescales (eg, from hourly to daily and weekly) are associated with the outcome from a theoretical and empirical standpoint. Most of the previous literature examining smartphone behavior and depression used a daily or hourly timescale to aggregate the behavioral measures [9,10,12]. Given this scarcity of discussion and recommendation, we chose to compute several behavioral measures (eg, app count, app entropy, session duration, and typing speed) at the hourly level, as the hourly level provides more temporal resolution about human-smartphone interaction behaviors. Future analysis can investigate the multiple timescales of behavioral measures and their associations with depressive symptoms.

Lastly, the sample size per person in this sample was a challenge owing to the nature of depression. Since the experience of loss of interest is likely to affect individuals’ motivation to complete their self-reports, it is a challenge to collect intensive longitudinal data on days when depressive symptoms are high. To allow the discovery of meaningful within-person associations, the number of self-reports per person is needed but is often scarce. This is one of the reasons why we selected only individuals whose survey compliance was high. Future studies need to develop creative ways to collect depressive symptom data, mitigate the missing data problem by imputation, and incorporate clinicians’ assessments of patients’ depressive symptoms.

### Conclusion

With the potential of using passive sensing as a low-burden method to identify mental health risks, research studies have developed and identified behavioral measures to assess the risk of elevated depressive symptoms. We proposed a set of behavioral measures from human-smartphone interactions based on theories of depression and previous empirical literature, and subsequently found evidence that app usage was associated with loss of interest in pleasurable activities, while typing speed and session duration were associated with depressed mood. We also highlighted the methodological consideration of disaggregating within- and between-person effects in longitudinal analysis, provided evidence to support how these 2 effects differ, and discussed the risks of not disaggregating them in the analysis. There are important open questions regarding the theoretical and methodological issues in this area, and we expect that the pursuit of these questions will advance the scientific discoveries of behavioral sensing studies in the coming years.

## Abbreviations

DSM: Diagnostic and Statistical Manual of Mental Disorders
DSM L1: Diagnostic and Statistical Manual of Mental Disorders Fifth Edition Self-Rated Level 1 Cross-Cutting Symptom Measure-Adult Survey
HIPAA: Health Insurance Portability and Accountability Act
PHQ2: short-form Patient Health Questionnaire

## References

[1] (2013). Diagnostic and Statistical Manual of Mental Disorders, Fifth Edition.

[2] Zarate D, Stavropoulos V, Ball M, de Sena Collier G, Jacobson NC (2022). Exploring the digital footprint of depression: a PRISMA systematic literature review of the empirical evidence. BMC Psychiatry.

[3] Mohr DC, Shilton K, Hotopf M (2020). Digital phenotyping, behavioral sensing, or personal sensing: names and transparency in the digital age. NPJ Digit Med.

[4] Mohr DC, Zhang M, Schueller SM (2017). Personal sensing: Understanding mental health using ubiquitous sensors and machine learning. Annu Rev Clin Psychol.

[5] De Fruyt J, Sabbe B, Demyttenaere K (2020). Anhedonia in depressive disorder: A narrative review. Psychopathology.

[6] Rizvi SJ, Pizzagalli DA, Sproule BA, Kennedy SH (2016). Assessing anhedonia in depression: Potentials and pitfalls. Neurosci Biobehav Rev.

[7] Trøstheim M, Eikemo M, Meir R, Hansen I, Paul E, Kroll SL, Garland EL, Leknes S (2020). Assessment of anhedonia in adults with and without mental illness: A systematic review and meta-analysis. JAMA Netw Open.

[8] Snaith RP, Hamilton M, Morley S, Humayan A, Hargreaves D, Trigwell P (1995). A scale for the assessment of hedonic tone the Snaith-Hamilton Pleasure Scale. Br J Psychiatry.

[9] Nickels S, Edwards MD, Poole SF, Winter D, Gronsbell J, Rozenkrants B, Miller DP, Fleck M, McLean A, Peterson B, Chen Y, Hwang A, Rust-Smith D, Brant A, Campbell A, Chen C, Walter C, Arean PA, Hsin H, Myers LJ, Marks WJ, Mega JL, Schlosser DA, Conrad AJ, Califf RM, Fromer M (2021). Toward a mobile platform for real-world digital measurement of depression: User-centered design, data quality, and behavioral and clinical modeling. JMIR Ment Health.

[10] Meyerhoff J, Liu T, Kording KP, Ungar LH, Kaiser SM, Karr CJ, Mohr DC (2021). Evaluation of changes in depression, anxiety, and social anxiety using smartphone sensor features: Longitudinal cohort study. J Med Internet Res.

[11] Zhang Y, Folarin AA, Sun S, Cummins N, Vairavan S, Bendayan R, Ranjan Y, Rashid Z, Conde P, Stewart C, Laiou P, Sankesara H, Matcham F, White KM, Oetzmann C, Ivan A, Lamers F, Siddi S, Vilella E, Simblett S, Rintala A, Bruce S, Mohr DC, Myin-Germeys I, Wykes T, Haro JM, Penninx BW, Narayan VA, Annas P, Hotopf M, Dobson RJ, RADAR-CNS Consortium (2022). Longitudinal relationships between depressive symptom severity and phone-measured mobility: Dynamic structural equation modeling study. JMIR Ment Health.

[12] Opoku Asare K, Terhorst Y, Vega J, Peltonen E, Lagerspetz E, Ferreira D (2021). Predicting depression from smartphone behavioral markers using machine learning methods, hyperparameter optimization, and feature importance analysis: Exploratory study. JMIR Mhealth Uhealth.

[13] Palmgreen P (1984). Uses and gratifications: A theoretical perspective. Annals of the International Communication Association.

[14] Moshe I, Terhorst Y, Opoku Asare K, Sander LB, Ferreira D, Baumeister H, Mohr DC, Pulkki-Råback L (2021). Predicting symptoms of depression and anxiety using smartphone and wearable data. Front Psychiatry.

[15] Narziev N, Goh H, Toshnazarov K, Lee SA, Chung K, Noh Y (2020). STDD: Short-term depression detection with passive sensing. Sensors (Basel).

[16] Bai R, Xiao L, Guo Y, Zhu X, Li N, Wang Y, Chen Q, Feng L, Wang Y, Yu X, Xie H, Wang G (2021). Tracking and monitoring mood stability of patients with major depressive disorder by machine learning models using passive digital data: Prospective naturalistic multicenter study. JMIR Mhealth Uhealth.

[17] Jacobson NC, Weingarden H, Wilhelm S (2019). Using digital phenotyping to accurately detect depression severity. J Nerv Ment Dis.

[18] Niemeijer K, Mestdagh M, Kuppens P (2022). Tracking subjective sleep quality and mood with mobile sensing: Multiverse study. J Med Internet Res.

[19] Giancardo L, Sánchez-Ferro A, Butterworth I, Mendoza CS, Hooker JM (2015). Psychomotor impairment detection via finger interactions with a computer keyboard during natural typing. Sci Rep.

[20] Vesel C, Rashidisabet H, Zulueta J, Stange JP, Duffecy J, Hussain F, Piscitello A, Bark J, Langenecker SA, Young S, Mounts E, Omberg L, Nelson PC, Moore RC, Koziol D, Bourne K, Bennett CC, Ajilore O, Demos AP, Leow A (2020). Effects of mood and aging on keystroke dynamics metadata and their diurnal patterns in a large open-science sample: A BiAffect iOS study. J Am Med Inform Assoc.

[21] Zulueta J, Piscitello A, Rasic M, Easter R, Babu P, Langenecker SA, McInnis M, Ajilore O, Nelson PC, Ryan K, Leow A (2018). Predicting mood disturbance severity with mobile phone keystroke metadata: A biaffect digital phenotyping study. J Med Internet Res.

[22] Snijders TAB, Bosker RJ (2011). Multilevel Analysis: An Introduction to Basic and Advanced Multilevel Modeling.

[23] Bolger N, Laurenceau JP (2013). Intensive Longitudinal Methods: An Introduction to Diary and Experience Sampling Research.

[24] Curran PJ, Bauer DJ (2011). The disaggregation of within-person and between-person effects in longitudinal models of change. Annu Rev Psychol.

[25] Bastiaens L, Galus J (2018). The DSM-5 self-rated level 1 cross-cutting symptom measure as a screening tool. Psychiatr Q.

[26] Kroenke K, Spitzer R, Williams JB (2003). The Patient Health Questionnaire-2 Validity of a two-item depression screener. Medical Care.

[27] Molenaar PCM (2004). A manifesto on psychology as idiographic science: Bringing the person back into scientific psychology, this time forever. Measurement: Interdisciplinary Research & Perspective.

[28] Bates D, Mächler M, Bolker B, Walker S (2015). Fitting linear mixed-effects models using lme4. J. Stat. Soft.

[29] Laukka EJ, Dykiert D, Allerhand M, Starr JM, Deary IJ (2017). Effects of between-person differences and within-person changes in symptoms of anxiety and depression on older age cognitive performance. Psychol. Med.

[30] Kroenke K, Spitzer RL, Williams JBW (2001). The PHQ-9: validity of a brief depression severity measure. J Gen Intern Med.

[31] Onnela J, Dixon C, Griffin K, Jaenicke T, Minowada L, Esterkin S, Siu A, Zagorsky J, Jones E (2021). Beiwe: A data collection platform for high-throughput digital phenotyping. Journal of Open Source Software.

[32] Torous J, Wisniewski H, Bird B, Carpenter E, David G, Elejalde E, Fulford D, Guimond S, Hays R, Henson P, Hoffman L, Lim C, Menon M, Noel V, Pearson J, Peterson R, Susheela A, Troy H, Vaidyam A, Weizenbaum E, Naslund JA, Keshavan M (2019). Creating a digital health smartphone app and digital phenotyping platform for mental health and diverse healthcare needs: an interdisciplinary and collaborative approach. J. Technol. Behav. Sci.

[33] Lind MN, Byrne ML, Wicks G, Smidt AM, Allen NB (2018). The Effortless Assessment of Risk States (EARS) Tool: An interpersonal approach to mobile sensing. JMIR Ment Health.

[34] Onnela J (2021). Opportunities and challenges in the collection and analysis of digital phenotyping data. Neuropsychopharmacology.

[35] Huckvale K, Venkatesh S, Christensen H (2019). Toward clinical digital phenotyping: a timely opportunity to consider purpose, quality, and safety. NPJ Digit Med.

[36] Müller SR, Peters H, Matz SC, Wang W, Harari GM (2020). Investigating the relationships between mobility behaviours and indicators of subjective well–being using smartphone–based experience sampling and GPS tracking. Eur J Pers.

[37] Wu X, Tao S, Zhang Y, Zhang S, Tao F (2015). Low physical activity and high screen time can increase the risks of mental health problems and poor sleep quality among Chinese college students. PLoS One.

